# Health-related quality of life and disability in patients with acute unilateral peripheral vestibular disorders^☆^

**DOI:** 10.1016/j.bjorl.2016.08.004

**Published:** 2016-08-24

**Authors:** Maria Petri, Magdalena Chirilă, Sorana D. Bolboacă, Marcel Cosgarea

**Affiliations:** a Iuliu Hatieganu University of Medicine and Pharmacy, Department of Otorhinolaryngology, Cluj-Napoca, Romania; b Iuliu Haţieganu University of Medicine and Pharmacy, Department of Medical Informatics and Biostatistic, Cluj-Napoca, Romania

**Keywords:** Unilateral peripheral vestibular disorders, DHI, SF-36, HRQoL, Distúrbios vestibulares periféricos unilaterais, DHI, SF-36, QVRS

## Abstract

**Introduction:**

Health-related quality of life is used to denote that portion of the quality of life that is influenced by the person's health.

**Objectives:**

To compare the health-related quality of life of individuals with vestibular disorders of peripheral origin by analyzing functional, emotional and physical disabilities before and after vestibular treatment.

**Methods:**

A prospective, non randomized case-controlled study was conduced in the ENT Department, between January 2015 and December 2015. All patients were submitted to customize a 36 item of health survey on quality of life, short form 36 health survey questionnaire (SF-36) and the Dizziness Handicap Inventory for assessing the disability. Individuals were diagnosed with acute unilateral vestibular peripheral disorders classified in 5 groups: vestibular neuritis, Ménière Disease, Benign Paroxysmal Positional Vertigo, cochlear-vestibular dysfunction (other than Ménière Disease), or other type of acute peripheral vertigo (as vestibular migraine).

**Results:**

There was a statistical significant difference for each parameter of Dizziness Handicap Inventory score (the emotional, functional and physical) between the baseline and one month both in men and women, but with any statistical significant difference between 7 days and 14 days. It was found a statistical significant difference for all eight parameters of SF-36 score between the baseline and one month later both in men and women; the exception was the men mental health perception. The correlation between the Dizziness Handicap Inventory and the SF-36 scores according to diagnostics type pointed out that the Spearman's correlation coefficient was moderate correlated with the total scores of these instruments.

**Conclusion:**

The Dizziness Handicap Inventory and the SF-36 are useful, proved practical and valid instruments for assessing the impact of dizziness on the quality of life of patients with unilateral peripheral vestibular disorders.

## Resumo

**Introdução:**

Qualidade de vida relacionados à saúde é utilizada para designar a parte da qualidade de vida que é influenciada pela saúde do indivíduo.

**Objetivos:**

Comparar a qualidade de vida relacionada à saúde de indivíduos com distúrbios vestibulares de origem periférica, analisando incapacidades funcionais, emocionais e físicas antes e após o tratamento vestibular.

**Método:**

Um estudo de caso-controle prospectivo, não randomizado foi conduzido no Departamento de Otorrinolaringologia, entre janeiro de 2015 e dezembro de 2015. Todos os pacientes foram submetidos a uma pesquisa de saúde personalizada de 36 itens sobre qualidade de vida, ao formulário abreviado de avaliação de saúde 36 (SF-36) e ao *Dizziness Handicap Inventory* para avaliar a incapacidade. Os indivíduos foram diagnosticados com distúrbios vestibulares periféricos unilaterais agudos, classificados em 5 grupos: neurite vestibular, Doença de Ménière, vertigem posicional paroxística benigna, disfunção cócleo-vestibular (exceto Doença de Ménière), ou outro tipo de vertigem periférica aguda (como enxaqueca vestibular).

**Resultados:**

Houve uma diferença estatisticamente significante para cada parâmetro de escore no *Dizziness Handicap Inventory* (emocional, funcional e físico) entre a avaliação basal e depois de um mês, tanto em homens quanto em mulheres, mas sem diferença estatística significativa entre 7 dias e 14 dias. Foi encontrada uma diferença estatisticamente significante para todos os oito parâmetros do escore no SF-36 entre a avaliação basal e um mês mais tarde, tanto em homens quanto em mulheres; a exceção foi a percepção de saúde mental nos homens. A correlação entre *Dizziness Handicap Inventory* e o SF-36 de acordo com o tipo de diagnóstico mostrou que o coeficiente de correlação de Spearman foi moderado quando correlacionado com o escore total desses instrumentos.

**Conclusão:**

O *Dizziness Handicap Inventory* e o SF-36 demonstraram ser instrumentos úteis, práticos e válidos para avaliar o impacto da tontura na qualidade de vida de pacientes com distúrbios vestibulares periféricos unilaterais.

## Introduction

Quality of life is defined as an individual perception of her/his position in life in the context of the culture and value systems in which she/he lives, and it encompasses a broad spectrum of domains including health status, economic resources, work status, relationships, and leisure activities.^1^ Health-Related Quality of Life (HRQoL) is used to denote that portion of the quality of life that is influenced by the person's health.

Vertigo or dizziness is one of the most common conditions that brings patients to emergency and its incidence increases with age.^2^ Despite a lifetime prevalence of dizziness and vertigo estimated at 20–30% and 1-year prevalence estimate for vertigo of 4.9%, the healthcare burden of vertigo is still relatively under-reported due to the unpredictability of attacks and the nature of the disease.^3^ An individual's progress or lack of progress in vestibular rehabilitation is usually measured by observing changes in motion intolerance, balance, functional abilities and more recently, health-related quality of life.^4^ Currently available conventional diagnostic tests (i.e. bed-side vestibular examination, videonystagmography, caloric and rotational tests, and posturography) are inadequate for evaluating the debilitating effects associated with vestibular disorders.^5^ Consequently, various questionnaires (such as General Depression Scale, Strait-Trait Anxiety Index, Dynamic Gait Index, the Functional Gait Assessment, the Balance Error Scoring System)^6^ have been developed in an attempt to quantify the self-perceived health status in vestibular patients with dizziness and imbalance.

The Dizziness Handicap Inventory16 (DHI) and the Medical Outcomes Study (MOS) 36-Item Short-Form Health Survey (SF-36) are two commonly used health-related quality-of life survey instruments. The SF-36 is a generic questionnaire with a global approach to measure health status as it relates to an individual's functional well-being; it has been widely used in studies of the outcome for chronic diseases, and it facilitates comparison of the clinical health of patients with the average standardized scores of the general population.^7^ The Dizziness Handicap Inventory (DHI), a disease-specific questionnaire, was developed for individuals with dizziness or balance problems and measures how vertigo and disequilibrium (imbalance) affect an individual's quality of life; this is a 25 item scale that was designed to evaluate the effect of dizziness and unsteadiness on the functional, emotional, and physical aspects of everyday life.^8^ The ability to detect the minimal clinical change in a variable over time is responsiveness; the DHI and the SF-36 provide the opportunity to compare the responsiveness of these two measurement instruments in HRQoL of patients with vestibular dysfunctioning.^9^

The aim of this study was to determinate the relationship between disability and HRQoL in patients with acute unilateral vertigo of peripheral origin by analyzing functional, emotional, and physical disabilities before and after vestibular treatment.

## Methods

A prospective, non randomized study was conducted on individuals diagnosed with acute unilateral vestibular peripheral disorders. All patients were submitted to customize a 36 item of health survey on quality of life, short form 36 health survey questionnaire (SF-36) and the Dizziness Handicap Inventory (DHI) for assessing the disability. All patients answered the SF-36 and DHI both at the beginning and the end of the follow up (SF-36: baseline & 1 month follow-up; DHI: baseline, 7 days, 14 days and 1 month follow-up). The study was approved by the Ethics Committee of University of Medicine and Pharmacy (Reference n° 200/8.05.2015).

The unilateral vestibular peripheral disorders were classified by type of peripheral vestibular disturbance in 5 groups: vestibular neuritis, Ménière disease, Benign Paroxysmal Positional Vertigo (BPPV), cochlear-vestibular dysfunction (other than Ménière disease), or other type of acute peripheral vertigo (as vestibular migraine).

Vestibular neuritis was diagnosed as an acute, sustained dysfunction of unilateral peripheral vestibular system with secondary nausea, vomiting, vertigo, and preserved auditory function. Symptoms typically started with a sudden attack of vertigo (a sensation of swaying or spinning) or dizziness, often accompanied by difficulty with vision or balance and could range from mild dizziness to a violent spinning or swaying sensation. The most severe symptoms generally lasted for a matter of days. Less-severe symptoms gradually diminished over several weeks, and some people recovered completely.

A typical attack of Meniere's disease was preceded by fullness in one ear; rarely occurred hearing fluctuation or changes in tinnitus. A Meniere's episode or “attack” generally involved severe vertigo (spinning), imbalance, nausea and vomiting as well as acute reduction of hearing. The average attack lasted two to four hours.

BPPV included dizziness or vertigo, lightheadedness, imbalance, and nausea. Activities which bring on symptoms will vary among persons, but in our patients symptoms were almost always precipitated by a change of position of the head with respect to gravity.

Cochlear-vestibular dysfunction included patients with acute vertigo of peripheral origin associated hearing loss or acute tinnitus who were not eligible for Meniere disease group.

Other type of acute peripheral vertigo refers to vertigo associated migraine. The clinical presentation of vestibular symptoms that often correlate with migraine included: dizziness; motion intolerance with respect to head, eyes, and/or body; spontaneous vertigo attacks (often accompanied by nausea and vomiting); diminished eye focus with photosensitivity; sound sensitivity and tinnitus; balance loss and ataxia; cervicalgia (neck pain) with associated muscle spasms in the upper cervical spine musculature; confusion with altered cognition; spatial disorientation; and anxiety/panic.

These subjects attended the ENT Department, Emergency County Hospital, between January 2015 and December 2015. The patients signed an informed consent form and accepted both the participation and the follow-up schedule prior to enrolment. All the patients were submitted to a complete ENT evaluation and complete medical history, neurological examination, otological and audiological assessment with impedance measures and auditory evoked potentials, vestibular tests, including videonystagmography, caloric tests, computer tomography or magnetic resonance imaging as appropriate. There were excluded: individuals who presented central vestibular alterations; “dizziness” without a diagnosis of acute unilateral peripheral vestibular disorder; people with clinical diagnosis of neurological degenerative diseases and/or cerebellar disease or posttraumatic vertigo; patients received prior vestibular rehabilitation or currently receiving vestibular rehabilitation, or using antivertiginous medication before presentation. Information was collected as demographic characteristics (age, gender, origin) and clinical data (type of peripheral vestibular disorder: BPPV, Ménière, other cochlear-vestibular dysfunction, vestibular neuritis, and other types of acute peripheral vertigo), use of medication (yes or no) and associated comorbidities.

The Short-Form Health Survey (SF-36) is a validated short-form health questionnaire composed of 36 items.^10^ SF-36 measures health on eight multi-item dimensions, covering functional status, well being, and overall evaluation of health: functional status with physical functioning (10 items), social functioning (2 items), role limitations (physical problems) (4 items), role limitations (emotional problems) (3 items), wellbeing with mental health (5 items), vitality (4 items), pain (2 items) and overall evaluation of health with general health perception (6 items). For each variable, item scores are coded, summed, and transformed on a scale from 0 (worst possible health state measured by the questionnaire) to 100 (best possible health state). It is a generic health measure, rather than disease-specific.

**Figure 1:**
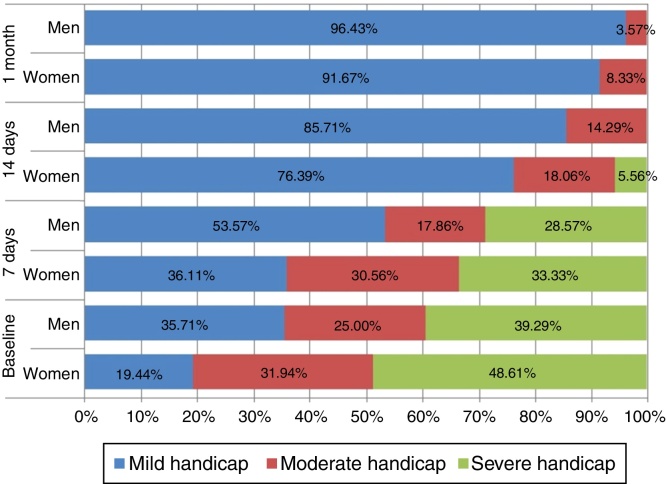
Disability function of the acute unilateral vestibular disorder according to gender.

DHI was developed by Jacobson et al. in 1990^6^ a tool used to quantify the impact of vertigo in daily life activities and situations. The Dizziness Handicap Inventory (DHI) is a validated 25 item, self-perceived handicap scale designed to assess the effect of dizziness and unsteadiness on quality of life.^10^ The items are grouped into 3 subscales evaluating the effects of dizziness and unsteadiness on emotional, functional, and physical aspects of daily living. For each item, there are 3 possible answers: no, sometimes, and yes, giving, respectively, 0, 2, and 4 points. There are seven questions assessing physical aspects, nine assessing emotional issues and nine evaluating functional issues. A total score of 100 corresponds to the worst self-perceived disability handicap, and a total score of 0 to the absence of handicap. As it might not be adequate to use the 3 subscales separately, only the total DHI score was used for interpretation. The questionnaires were submitted to patients during an interview at the ENT department and the data were collected for calculating the disability in function of the acute unilateral vestibular disorder according to gender (Fig. 1).

All statistical tests were performed with Statistica software, version 8.0 (StatSoft, Inc. 2300 East 14th Street, Tulsa, OK 74104). Categorical variables were summarized with percentage and associated 95% Confidence Intervals computed using an exact formula.^11^ Group percentages were compared with the use of *Z* test for proportions. Numerical variables were summarized as mean ± standard deviation whenever data proved to follow normal distribution and median and IRQ (Q1–Q3), where Q1 = 25th percentile, Q3 = 75th percentile) otherwise. Two group means for normal variables and non-normal distribution were compared with the used of Student *t*-test for independent groups, paired Student *t*-test, and the Mann–Whitney *U*-test, respectively. Friedman ANOVA test was applied to compare longitudinal numerical data non-normal distributed on dependent samples. Two-tailed *p*-value less than 0.05 indicated statistical significance.

## Results

One hundred subjects completed the study (baseline and follow-up evaluations), with significant higher number of women compared to men (72% [62.01–80.99] vs. 28% [19.01–37.99]; *Z*-statistic = −9.80, *p* < 0.0001). The age of patients ranged from 28 to 71 years old (47.78 ± 14.74 years), without a significant difference (*t*-statistic = −0.6501, *p* = 0.5172) between women (47.18 ± 14.91) and men (49.32 ± 14.46). For both genders, the majority of patients were aged 51–60 years old (29.17% [19.46–41.65] women vs. 39.29% men [21.56–60.59]), then 31–40 years old (20.83% [12.52–31.92] women vs. 25.00% men [10.84–46.3]). A significant (*Z*-statistic = −12.83, *p* < 0.0001) higher percent of patients were from urban areas (77% [68.01–84.99]) compared to rural areas (23% [15.01–31.99]).

On both genders, the most frequent diagnosis was vestibular neuritis. A significant higher percent of women were diagnosed with Ménière disease compared to men (11.11% vs. 3.57%; *Z*-statistic = −2.40, *p* = 0.0164), without other significant differences between genders (*p* > 0.05).

**Figure 2:**
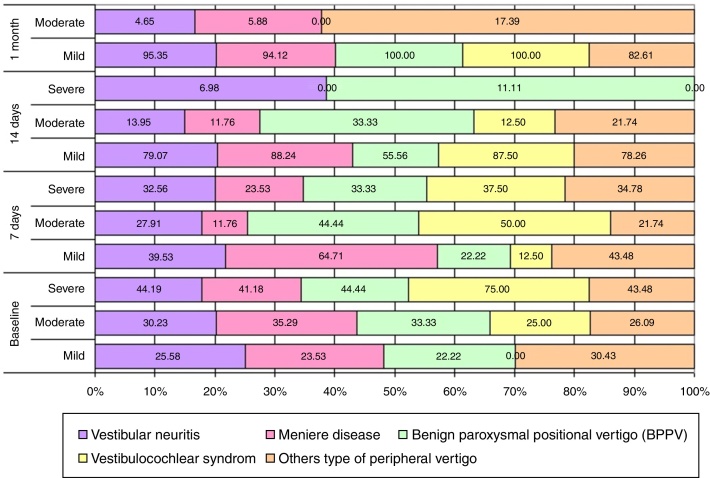
Handicap according with DHI and diagnosis.

Classification of the handicap according with DHI and diagnosis over the time is presented in Fig. 2.

Within each DHI domains of dizziness, significant differences have been observed between pairs of investigated domains on both women and men (see Friedman ANOVA in Table 1, *p* < 0.0001). There was a statistical significant difference for each parameter of DHI score (the emotional, functional and physical) between the baseline and one month (Table 1) both in men and women, but with any statistical significant difference between 7 days and 14 days.

**Table 1:** DHI scores at the baseline, 7 and 14 days, and 1 month.

Domain	Women	Men	Mann–Whitney (*p*-value)
*Emotional*			
Baseline	13 (6–20)	15 (6–22)	1.15 (0.2481)
7 days	10 (4–18)	11 (4–18)	1.07 (0.2824)
14 days	6 (0–10)	8 (2–12.5)	0.66 (0.5061)
1 month	2 (0–4)	5 (2–8.5)	0.77 (0.4439)
Friedman ANOVA (*p*-value)	183.83 (<0.0001)	65.53 (<0.0001)	
Kendall Concordance	0.849	0.772	

*Functional*			
Baseline	22 (14–28)	21 (16–29)	1.16 (0.2452)
7 days	17 (10–24)	18 (15–26)	1.61 (0.1083)
14 days	8 (4–12)	11 (7.5–17)	1.80 (0.0714)
1 month	4 (2–8)	8 (4–12.5)	0.98 (0.3267)
Friedman ANOVA (*p*-value)	205.44 (<0.0001)	73.96 (<0.0001)	
Kendall Concordance	0.9504	0.8671	

*Physical*			
Baseline	18 (12–22.5)	17 (12–22.5)	1.05 (0.2959)
7 days	14 (8–20)	14 (9.5–18.5)	1.33 (0.1849)
14 days	6 (4–10)	8 (3.5–12)	0.79 (0.4278)
1 month	2 (0–4)	4 (2–8)	−0.49 (0.6213)
Friedman ANOVA (*p*-value)	203.03 (<0.0001)	69.61 (<0.0001)	
Kendall Concordance	0.9391	0.8223	

*Total*			
Baseline	51 (35.5–70)	47 (39–70)	1.26 (0.2064)
7 days	42 (26–58.5)	43 (29.5–59)	1.68 (0.0938)
14 days	22 (10–32)	24 (17.5–42)	1.47 (0.1410)
1 month	10 (4–18.5)	17 (10–27)	0.54 (0.5899)
Friedman ANOVA (*p*-value)	210.92 (<0.0001)	77.03 (<0.0001)	
Kendall Concordance	0.9762	0.914	

Within each SF-36 domains, significant differences have been observed between pairs of investigated domains on both women and men, unless in mental health at one month in men (see Mann–Whitney test, Paired Student *t*-test in Table 2, *p* < 0.0001). It was found a statistical significant difference for all eight parameters of SF-36 score between the baseline and one month later both in men and women; the exception was the men mental health perception with no statistical significant difference between the baseline and one month later (Student *t*-test, *p* = 0.1966) (Table 2).

**Table 2:** SF-36 scores at the baseline and 1 month.

Domain	All	Women	Men	*p*-value
*Physical Health- Physical Functioning (PF)*^a^				
Baseline	550.0 (350.0–750.0)	550.0 (337.5–550.0)	625.0 (387.5–787.5)	−0.74 (0.4611)
1 month	900.0 (800.0–1000.0)	900.0 (800.0–900.0)	900.0 (700.0–962.5)	0.26 (0.7970)
Statistics (*p*-value)	9.70 (<0.0001)	8.19 (<0.0001)	5.00 (<0.0001)	

*Role-Physical (RP)*^a^				
Baseline	0.0 (0.0–200.0)	0.0 (0.0–0.0)	0.0 (0.0–200.0)	0.10 (0.9205)
1 month	400.0 (400.0–400.0)	400.0 (400.0–400.0)	400.0 (75.0–400.0)	0.83 (0.4070)
Statistics (*p*-value)	7.88 (<0.0001)	6.72 (<0.0001)	3.88 (0.0001)	

*Bodily Pain (BP)*^a^				
Baseline	90.0 (65.0–135.0)	90.0 (65.0–90.0)	112.5 (88.8–136.3)	−0.95 (0.3431)
1 month	140.0 (135.0–180.0)	140.0 (130.0–140.0)	155.0 (135.0–185.0)	−0.33 (0.7413)
Statistics (*p*-value)	9.06 (<0.0001)	7.75 (<0.0001)	4.51 (<0.0001)	

*General Health (GH)*^b^				
Baseline	276.00 ± 102.37	272.92 ± 105.13	283.93 ± 96.28	−0.48 (0.6315)
1 month	417.25 ± 65.20	414.93 ± 65.90	423.21 ± 64.16	−0.57 (0.5710)
Statistics (*p*-value)	−17.08 (<0.0001)	−13.76 (<0.0001)	−10.55 (<0.0001)	

*Mental Health Vitality (VT)*^a^				
Baseline	200.0 (120.0–245.0)	200.0 (140.0–200.0)	210.0 (120.0–245.0)	−0.02 (0.9816)
1 month	320.0 (280.0–340.0)	320.0 (280.0–320.0)	320.0 (280.0–345.0)	−0.45 (0.6561)
Statistics (*p*-value)	9.80 (<0.0001)	8.25 (<0.0001)	5.10 (<0.0001)	

*Role-Emotional (RE)*^a^				
Baseline	0.0 (0.0–300.0)	0.0 (0.0–300.0)	100.0 (0.0–300.0)	−1.21 (0.2281)
1 month	300.0 (300.0–300.0)	300.0 (300.0–300.0)	300.0 (300.0–300.0)	−0.27 (0.7882)
Statistics (*p*-value)	7.08 (<0.0001)	6.18 (<0.0001)	3.18 (0.0015)	

*Social Functioning (SF)*^a^				
Baseline	100.0 (75.0–125.0)	100.0 (75.0–100.0)	87.5 (75.0–162.5)	−0.40 (0.6897)
1 month	175.0 (150.0–200.0)	175.0 (150.0–175.0)	175.0 (150.0–200.0)	−1.17 (0.2417)
Statistics (*p*-value)	9.38 (<0.0001)	8.19 (<0.0001)	4.36 (<0.0001)	

*Mental Health (MH)*^b^				
Baseline	341.20 ± 70.89	335.28 ± 73.50	356.43 ± 62.31	−1.35 (0.1817)
1 month	355.00 ± 66.14	350.83 ± 67.21	365.71 ± 63.21	−1.01 (0.3148)
Statistics (*p*-value)	−3.24 (0.0016)	−2.95 (0.0043)	−1.32 (0.1966)	

a Median (interquartile range expressed as Q1–Q3), Sign test on row comparison between baseline and 1 month) & Mann–Whitney test on the last column (comparisons between women and men).

b Mean ± standard deviation, Paired Student *t*-test on row (comparison between baseline and 1 month) & Independent Samples *t*-test on last column (comparisons between women and men).

The functional, emotional, and physical pre-treatment scores were significantly higher in patients with MD than in patients with vestibular neuritis or BPPV (which implies that inconvenience that patients with MD face in their daily life appears to be the greatest; (Spearman Rank Correlation, *p* < 0.05). The correlation between DHI and SF-36 scores according to diagnostics type pointed out that the Spearman's correlation coefficient was moderate correlated with the total scores of both instruments. The negative values indicate score inversions in the answers of one instrument relative to the other (Table 3).

**Table 3:** Correlations between DHI and SF-36 scores according to diagnosis (*n* = 100).

Pairs of variables	Spearman correlation coefficient	*p*-level
E & PF	−0.4039	<0.0001
E & RP	−0.3498	0.0004
E & RE	−0.4129	<0.0001
E & MHV	−0.3333	0.0007
E & MH	−0.4034	<0.0001
E & SF	−0.4240	<0.0001
E & BP	−0.2939	0.0030
E & GH	−0.2511	0.0117
FU & PF	−0.5529	<0.0001
FU & RP	−0.3697	0.0002
FU & RE	−0.3766	0.0001
FU & MHV	−0.2610	0.0087
FU & MH	−0.3877	0.0001
FU & SF	−0.3310	0.0008
FU & BP	−0.3313	0.0008
FU & GH	−0.2868	0.0038
PH & PF	−0.5289	0.0000
PH & RP	−0.2512	0.0117
PH & RE	−0.2502	0.0120
PH & MHV	−0.1548	0.1241
PH & MH	−0.2863	0.0039
PH & SF	−0.2061	0.0397
PH & BP	−0.2895	0.0035
PH & GH	−0.1500	0.1363
Total & PF	−0.5830	<0.0001
Total & RP	−0.3948	<0.0001
Total & RE	−0.4242	<0.0001
Total & MHV	−0.3045	0.0021
Total & MH	−0.4484	<0.0001
Total & SF	−0.3894	<0.0001
Total & BP	−0.3531	0.0003
Total & GH	−0.2819	0.0045

DHI variables: E, emotional; FU, functional; PH, physical.

SF-36 variables: PF, Physical Functioning; RP, Role Physical; RE, Role Emotional; MHV, Mental Health Vitality; MH, Mental Health; SF, Social Functioning; BP, Bodily Pain; GH, General Health.

## Discussion

The objective of this research was to determine the relationship between disability and HRQoL in patients with acute unilateral vertigo of peripheral origin before and after vestibular treatment. Besides the correlation obtained in the majority of the responses provided by participants, the variables of both questionnaires showed an inverse behavior: higher deterioration in the quality of life expressed by low scores was correlated with higher degree of disability expressed by high scores, and vice versa.

In our study the higher improvement in total DHI score and SF-36 scales at one month after treatment was for patients with vestibular neuritis and BPPV, two well-treatable conditions. We also observed a good quality improvement of DHI total score for Ménière disease after one month compared with other cochlear-vestibular syndromes or vestibular migraine. We have to specify that we performed solumedrol transtympanic injections for all patients with MD with very good results on acoustic symptoms and good for vertigo; another possible explanation for these results could be the pathophysiology of MD as inner ear disease is much conceivable to the individual patient than migraine as rather complicated brain disorder.^5^ Correlations between internal categories of both questionnaires used in our study shown that most of them presented moderate relationships. The three scales of the SF-36 that were most related to mental health had correlations with the DHI emotional score.

In the literature, the type of peripheral vestibular disorder that occurs more frequently is benign Paroxysmal Positional Vertigo (BPPV).^12^ We found that the most frequent diagnosis on both genders was vestibular neuritis.

In our study, 72% of samples were women that coincide with authors that had described the prevalence of vestibular symptoms in women for several reasons such as menopause, osteoporosis, cardio-vascular and metabolic diseases, which cause symptoms of dizziness.^13^ However, Obermann et al.^5^ considered that selection bias of older women may have influenced the study results and, consequently, the interpretation.

Like in our case-controlled study, the samples used in previous studies also differed in the etiology of dizziness and unsteadiness.^14^, ^15^, ^16^ For example, patients with Ménière's disease typically suffer from unpredictable attacks of symptoms and have a high comorbidity of anxiety and depression disorders, and as a result, frequently develop avoidance behavior that can lead to limitations in their participation.

HRQOL measurement varies among individuals because it is a dynamic construct containing continuous interactions between the patients and their environment, and includes physical, mental and social aspects.^17^, ^18^ Consequently, it is appropriate to examine the interaction between the diseases, the changes occurring in the life of the individual, the received and perceived social contribution and the stage of life at which the pathology takes place. Most studies on BPPV report a good efficacy of BPPV treatment within first week, but Obermann et al. pointed out very good results after 2 years follow-up.^5^ In a study involving 25 patients with dizziness and diagnostic suspicion of peripheral vestibular syndrome, Ganança et al.^19^ showed that patients with chronic dizziness had a worse quality of life in terms of physical, functional and emotional aspects of their lives seen on the Brazilian DHI.

Our results showed better responses at one month with any statistical significant difference between 7 days and 14 days.

Evaluation of quality of life can be used in daily practices to measure contribution of clinical handling to diminish the impact of chronic diseases to daily routine of patients.

Our study has some limitations. First, the small number of patients could not offer a strong conclusion. Second, the selection bias (older women, exclusion of very lightly affected cases that were addressed to the general practitioners) may have an important influence on the results.

## Conclusion

The DHI and the SF-36 are useful, proved practical and valid instruments for assessing the impact of dizziness on the quality of life of patients with unilateral peripheral vestibular disorders. The relationship between disability and HRQoL according to diagnostics type pointed out a moderate correlation with the total scores of both instruments before and after vestibular treatment.

## Conflicts of interest

The authors declare no conflicts of interest.
